# Clinical Efficacy of Clear Aligners in Class II Malocclusion: From Pediatric to Adult Cases–A Narrative Review

**DOI:** 10.3390/jfb16090354

**Published:** 2025-09-19

**Authors:** Gianna Dipalma, Grazia Marinelli, Francesco Inchingolo, Marialuisa Longo, Maral Di Giulio Cesare, Sharon Di Serio, Andrea Palermo, Massimo Del Fabbro, Alessio Danilo Inchingolo, Angelo Michele Inchingolo

**Affiliations:** 1 Interdisciplinary Department of Medicine, University of Bari “Aldo Moro”, 70124 Bari, Italy; gianna.dipalma@uniba.it (G.D.); graziamarinelli@live.it (G.M.); dott.marialuisa.longo@gmail.com (M.L.); maraldgc@gmail.com (M.D.G.C.); sharondiserio954@gmail.com (S.D.S.); angelomichele.inchingolo@uniba.it (A.M.I.); 2 Department of Biomedical, Surgical and Dental Sciences, Milan University, 20122 Milan, Italy; massimo.delfabbro@unimi.it; 3 Department of Experimental Medicine, University of Salento, 73100 Lecce, Italy; andrea.palermo@unisalento.it; 4 Unit of Maxillo-Facial Surgery and Dentistry, Fondazione IRCCS Ca’ Granda Ospedale Maggiore Policlinico, 20122 Milan, Italy

**Keywords:** aligner, Class II, malocclusion, distalizzation

## Abstract

Background: Class II malocclusion is one of the most common and challenging orthodontic problems, often requiring complex, lengthy treatment and sometimes involving extractions or surgery. While conventional fixed appliances have been the gold standard, the increasing demand for aesthetic and comfortable treatment alternatives has made clear aligners a prevalent choice. Understanding the specific biomechanics, limitations, and successful clinical strategies for using aligners—especially in managing vertical dimension and achieving skeletal correction (mandibular advancement)—is crucial for expanding non-invasive treatment options and improving outcomes for a broad range of Class II patients. Objective: The objective of this review is to examine the effectiveness and clinical approaches of clear aligners in Class II correction across different age groups, with particular attention to vertical control, mandibular advancement methods, and the predictability of tooth movements in both growing and fully mature patients. Materials and Methods: This review narratively discusses the most relevant clinical findings and practical strategies for managing Class II malocclusions with clear aligners. Particular attention is given to the integration of auxiliary devices, such as elastics, attachments, and temporary anchorage devices (TADs), which can enhance biomechanical control. Results: The combination of aligners with mini-implants and attachments resulted in a consequent decrease in excessive overjet, improvement in facial profile, and long-term stability supported by fixed retention. In growing patients, correction benefited from mandibular advancement protocols and control of molar extrusion, allowing for preservation of the mandibular plane angle. Movement predictability showed higher reliability in anterior torque movements, whereas maxillary incisor intrusion remained less predictable. Conclusions: Clear aligners, especially when supported by auxiliary device, such as mini-implants and attachments, offer a reliable and aesthetic alternative to conventional orthodontic treatment for Class II malocclusions. However, certain tooth movements may still be less predictable, highlighting the need for careful planning, individualized biomechanics, and ongoing technological improvements.

## 1. Introduction

Class II malocclusions represent one of the most common orthodontic challenges worldwide, affecting approximately 15–20% of patients in industrialized countries [1,2,3,4,5]. These malocclusions are characterized by a distal relationship of the lower first molars relative to the upper first molars, often accompanied by increased overjet and a convex facial profile [6,7,8,9,10,11,12,13,14,15]. The etiology is multifactorial and includes genetic factors, skeletal discrepancies between the maxilla and mandible, unfavorable growth patterns, prolonged habits, such as thumb sucking, and functional problems like chronic mouth breathing [16,17,18,19,20,21,22,23,24,25,26].

Managing Class II malocclusions remains challenging, often requiring a combination of skeletal and dental strategies tailored to the individual patient [27,28,29,30,31,32,33,34,35,36]. Clinically, growth modification appliances can be effective in children, while adults may benefit from non-surgical orthodontic strategies or, in some cases, surgical interventions [37,38,39,40,41,42,43,44].

In recent years, advances in orthodontic technology and increasing patient demand for less invasive, more aesthetic treatments have driven the rapid adoption of clear aligners [45,46,47,48,49,50,51]. These removable, transparent devices are digitally designed and produced through CAD/CAM and 3D printing technologies [52,53,54,55,56,57]. Initially reserved for mild cases like minor crowding or spacing, improvements in materials, customized attachments, and the integration of intermaxillary elastics have expanded the clinical scope of aligners, making them a viable option for managing complex malocclusions, including Class II cases [58,59,60,61,62,63,64].

The key advantage of clear aligners in treating Class II malocclusions lies in their capacity for detailed digital planning [12,13,22,23,25,26]. This allows orthodontists to carefully program distalization of molars and canines, applying intermaxillary traction through elastics attached to the aligners, similarly to traditional fixed methods [65,66,67,68,69,70,71,72,73,74,75,76]. Attachments bonded to teeth enhance control over difficult movements, such as root rotations and selective intrusion or extrusion, thus increasing treatment predictability [77,78,79,80,81,82,83,84].

Clinical evidence suggests that clear aligners can be effective, especially in mild to moderate Class II cases or as a refinement tool after fixed appliance therapy [85,86,87,88]. Certain movements may still be challenging, especially large skeletal corrections, and require careful planning and patient monitoring [89,90,91,92,93,94,95]. Despite this, aligners offer significant benefits, including improved aesthetics, greater patient comfort, easier oral hygiene, and fewer emergency visits [96,97,98,99,100,101,102,103,104].

Patient compliance is critical, as aligners need to be worn consistently for 20–22 h daily to ensure effective tooth movement [105,106,107,108]. Digital simulation software enhances treatment planning and patient communication by providing visual progress predictions, though these are idealized and must be adjusted based on biological response [109,110,111,112,113,114,115,116,117].

Clinicians should consider costs, patient motivation, and realistic expectations when planning aligner therapy while continuing to refine strategies based on evolving clinical evidence [118,119,120,121,122,123,124,125,126,127].

Overall, treating Class II malocclusions with clear aligners demands a thorough understanding of orthodontic biomechanics, digital design skills, and active patient engagement [1,2,3,4,6,8,9,10,24,58]. This narrative review is intended to offer clinicians a concise and practical synthesis of contemporary strategies for managing Class II malocclusions with clear aligners [128,129,130,131,132,133,134]. Special emphasis is placed on both pediatric and adult patients, highlighting clinical considerations, treatment planning nuances, and emerging techniques aimed at optimizing dental and skeletal outcomes [135,136,137,138,139,140,141,142,143,144].

## 2. Materials and Methods

In this narrative review, we considered the most relevant and recent clinical evidence, including case reports and prospective studies, addressing the use of clear aligners in the management of Class II malocclusions.

### Search Process

**Figure 1:**
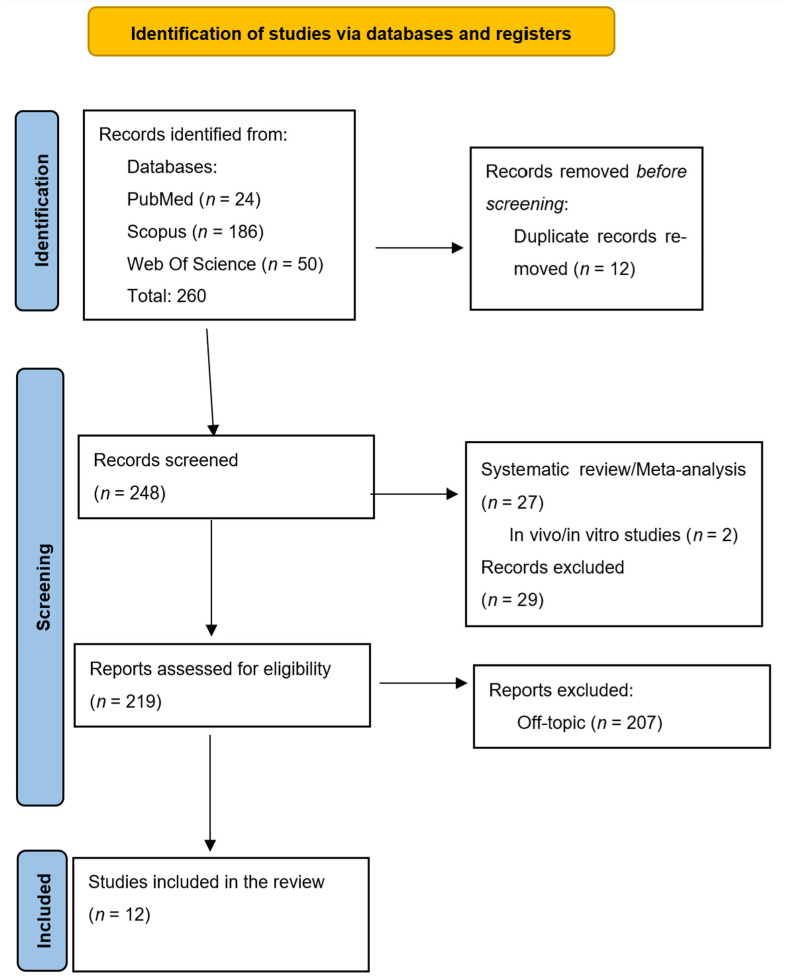
PRISMA flow chart of the literature search and article inclusion process.

The literature was searched mainly through PubMed, Scopus, and Web of Science, focusing on publications from the last decade. Particular attention was given to studies that directly addressed clear aligner treatment in patients with Class II malocclusion (Figure 1).

Rather than applying rigid inclusion or exclusion rules, we selected articles that provided clinically relevant information on the use of aligners for Class II correction. Both adolescent and adult populations were considered, and studies of different designs (clinical trials, case reports, prospective or retrospective analyses) were integrated when they contributed meaningful clinical insights.

The guiding aim of this review was to explore whether clear aligners, alone or combined with auxiliary devices, can provide effective correction of Class II malocclusions and to highlight the strengths and limitations reported in the available literature.

The studies identified were read in full and synthesized narratively, emphasizing recurring themes, treatment strategies, and clinical outcomes rather than strict methodological appraisal.

## 3. Results

The available literature on the use of clear aligners for the treatment of Class II malocclusions is still limited but growing. Among the studies published in the last decade, only a minority have directly addressed Class II correction, with considerable variability in patient age, treatment protocols, and diagnostic approaches. Overall, the evidence includes clinical trials, prospective and retrospective studies, as well as case reports. Despite methodological differences, these studies provide valuable insights into treatment outcomes, highlighting both the potential and the limitations of aligner therapy in managing skeletal and dental Class II malocclusions.

Synthesizing the collective evidence, clear aligners provide a balance of clinical efficacy, patient-centered advantages, and predictable biomechanical control. They reliably manage sagittal and vertical dimensions, effectively limit unwanted mandibular incisor proclination, and enhance patient compliance and satisfaction. Complex movements, such as torque, deep bite correction, and rotations, require careful planning, auxiliary integration, and staged refinement to optimize predictability. When applied thoughtfully, clear aligners leverage system-specific properties, digital planning, and biomechanical strategies to achieve outcomes that are functionally, aesthetically, and clinically robust. This synthesis underscores the necessity of meticulous planning, ongoing evaluation, and adaptive strategies to fully harness the potential of clear aligners, highlighting both their current strengths and avenues for future refinement and innovation in orthodontic practice.

### Quality Assessment and Risk of Bias of Included Articles

The quality and potential bias of the included studies were systematically evaluated using the RoB-2 tool, which provides a standardized assessment across several methodological domains: selection bias, performance bias, detection bias, attrition bias, and reporting bias. This evaluation was adapted according to the study designs, with randomized controlled trials receiving particular scrutiny for randomization and blinding, while observational studies were assessed for selection and confounding factors. Each study was classified with a risk level of low, moderate, or high for each bias domain. The software generated a color-coded matrix to visually summarize the risk of bias (Table 1). Overall, most clinical and prospective studies demonstrated low to moderate risk of bias, reflecting sound methodological rigor and appropriate controls. However, some retrospective studies and case reports were found to have higher susceptibility to bias, mainly due to limited sample sizes, lack of control groups, or incomplete outcome reporting.

## 4. Discussion

The theme of predictability in orthodontic tooth movement is further explored by Castroflorio et al. in a prospective study involving 79 patients. Their research highlighted that certain orthodontic movements remain challenging to predict accurately, even when treatment planning is performed by experienced clinicians. The study identified that the type and placement of attachments play a critical role in influencing the effectiveness and precision of these movements. Additionally, the material properties of the clear aligners themselves were found to significantly affect treatment outcomes. These findings carry important clinical implications, emphasizing that to achieve optimal results, practitioners must incorporate careful virtual overcorrections into their treatment plans. Moreover, thoughtful selection and customization of attachment design and positioning are essential to enhance aligner grip and force application, ultimately improving the accuracy and efficiency of tooth movement. This underscores the complexity of clear aligner therapy and the necessity of a meticulous, individualized approach in treatment planning.

When comparing aligners with traditional appliances, such as the pendulum, both approaches appear capable of correcting molar relationships and overjet. Yet, the difference lies in the vertical effects; while traditional appliances tend to cause extrusion and occlusal plane rotation, aligners—with their full-coverage design and “bite block effect”—offer superior vertical control. This makes them particularly advantageous in patients where stability of facial height and occlusal plane orientation are priorities.

In recent years, orthodontics has undergone a profound transformation, largely driven by the advent, progressive refinement, and widespread adoption of clear aligner therapy. Initially conceived as a treatment option reserved for cases of mild crowding and primarily aesthetic concerns, aligners have gradually demonstrated the potential to manage an increasingly broad spectrum of malocclusions. This expansion now includes not only moderate but also complex clinical scenarios that, until a few decades ago, were addressed almost exclusively with fixed multibracket appliances or even surgical approaches. The continuous evolution of aligner technology, together with improvements in digital planning software, materials, and auxiliary devices, has stimulated a rapidly growing body of scientific literature. Their success can be attributed to a unique combination of factors: improved aesthetics, enhanced comfort, and the convenience of being removable. Unlike conventional braces, aligners allow patients to maintain better oral hygiene, reduce dietary restrictions, and integrate treatment more seamlessly into their daily lives. This convergence of patient-centered benefits and technological innovation has positioned aligners not only as a cosmetic solution but also as a scientifically validated and widely accepted treatment modality in contemporary orthodontics. This body of work examines in depth the capabilities, limitations, and clinical nuances of clear aligners, paying particular attention to aspects such as the control of incisor inclination, the predictability of tooth movements, the correction of vertical discrepancies (for example, deep bites), the management of sagittal problems, such as Class II malocclusions, through molar distalization, and the integration of auxiliaries and biomechanical strategies to enhance performance.

Across these studies, a recurring observation emerges: while aligners undeniably provide important advantages in terms of patient comfort, aesthetics, and, in certain circumstances, even better control of specific dental movements compared with traditional appliances, their efficacy in producing complex or high-load biomechanical movements remains variable. This variability requires not only careful planning but also, in many cases, the adjunctive use of auxiliary systems or staged refinements to achieve predictable outcomes. As a consequence, aligner therapy is increasingly recognized not as a uniform, standardized modality but rather as a flexible, individualized strategy that demands both technical expertise and a solid understanding of biomechanics.

### 4.1. Control of Mandibular Incisor Proclination

Among the most consistently reported advantages of aligners is their superior control of mandibular incisor inclination. Excessive proclination of lower incisors, often observed with fixed appliances, is a clinically undesirable effect because it can increase the risk of periodontal recession, reduce alveolar bone support, and compromise long-term stability by predisposing to relapse. Hennessy and colleagues conducted a randomized clinical trial directly comparing conventional labial fixed appliances with clear aligners. Their findings showed a significant proclination of the lower incisors in the fixed appliance group, while aligners maintained a more favorable inclination pattern. This difference has considerable clinical importance, as periodontal preservation and stability of the anterior segment represent key treatment goals in contemporary orthodontics.

Similarly, Dianiskova and collaborators, studying growing patients with mild Class II malocclusions, confirmed that both treatment modalities—fixed appliances and aligners—achieved satisfactory sagittal correction. However, aligners exhibited a clear advantage in minimizing unwanted proclination of lower incisors. This feature, in addition to the greater patient-reported comfort and aesthetic satisfaction, further supports the clinical use of aligners in select cases.

These findings collectively reinforce the idea that aligners should not be regarded solely as a cosmetic alternative to fixed appliances. On the contrary, they offer tangible biomechanical advantages, especially in scenarios where maintaining incisor inclination within optimal limits is crucial. This is particularly relevant in patients with reduced periodontal support, a thin biotype, or situations where anterior anchorage control is paramount.

### 4.2. Deep Bite Correction and Vertical Movements

While aligners demonstrate reliable control in some dimensions, their predictability in vertical corrections, such as deep bite reduction, remains more controversial. Deep bite correction requires controlled intrusion of anterior teeth and/or extrusion of posterior segments, movements that are biomechanically demanding due to resistance from surrounding anatomical structures and the limitations of plastic materials.

Shahabuddin and colleagues, in a retrospective study of adult patients treated with Invisalign, found that only about one-third of the planned deep bite correction was actually achieved. The mean reduction in overbite was 1.15 mm compared with 3.35 mm predicted digitally. Interestingly, the discrepancy was particularly evident in upper incisor movements; planned intrusion frequently manifested instead as extrusion, highlighting the intrinsic biomechanical limitations of aligners in applying purely vertical forces.

Auxiliary elements, such as bite ramps or elastics, have been proposed to enhance vertical control. However, their effectiveness appears inconsistent, and in Shahabuddin’s study, their use did not yield statistically significant improvements. This evidence emphasizes the necessity of adopting strategies like planned overcorrections, refinement stages, and continuous clinical monitoring to achieve acceptable clinical outcomes.

Broader clinical experience supports these observations, as vertical control continues to represent one of the main challenges in aligner therapy. For this reason, careful case selection and biomechanical planning are essential, especially in adult patients where vertical discrepancies may be compounded by reduced periodontal support or skeletal patterns unfavorable to bite opening.

### 4.3. Torque Movements and Three-Dimensional Control

Another area of significant discussion concerns the ability of aligners to achieve torque control, that is, the buccolingual inclination of tooth roots. Achieving torque requires the application of forces at a distance from the center of resistance, which is particularly challenging with removable plastic appliances.

Rajan and colleagues evaluated the capacity of Invisalign to perform torque movements exceeding 10° in maxillary central incisors. Their study revealed that only about 42% of the planned torque was clinically expressed, with a high incidence of underexpression—often between 10° and 15° less than predicted. Moreover, the actual movement frequently manifested as uncontrolled tipping rather than true root torque, underscoring the difficulty in translating virtual digital planning into precise three-dimensional reality.

Interestingly, not all systems exhibit the same performance. Tepedino and collaborators reported that the Nuvola^®^ aligner system was capable of producing torque movements in the anterior region with high accuracy, achieving outcomes closely aligned with planned values. This contrast suggests that system-specific design features—including aligner stiffness, material resilience, precision in attachment design, and software algorithms—may significantly influence clinical effectiveness. Consequently, clinicians must be aware that not all aligner systems are interchangeable, and the characteristics of each product should be carefully considered when planning complex movements.

### 4.4. Class II Correction and Molar Distalization

The management of Class II malocclusions with clear aligners has been extensively investigated, especially with regard to molar distalization. Finite element modeling studies, such as those conducted by Y. Li and colleagues, have shown that the efficacy of molar distalization and its side effects on anterior teeth are influenced by multiple variables, including the stage of dental development, the direction of traction, and the type of auxiliary device employed. For example, auxiliaries, such as the Angel button, precision incision hooks, or lingual buttons, can modify the biomechanics of distalization, with bilateral distalization tending to increase anterior tipping and stress on periodontal tissues.

Comparative clinical research also supports these insights. Lione and collaborators analyzed distalization achieved with pendulum appliances versus clear aligners, finding that both modalities effectively corrected molar relationships and overjet. However, aligners demonstrated superior vertical control, limiting molar extrusion and occlusal plane rotation. This is attributable to their full-coverage design and bite-block effect, which distribute forces more evenly and counteract undesired vertical changes.

Prospective evaluations in growing patients, such as those by Balboni and colleagues, further demonstrated that sequential distalization with aligners not only achieved effective sagittal correction but also preserved vertical skeletal parameters, such as lower anterior facial height and mandibular plane angle. This preservation is of great clinical importance, as vertical dimension changes can significantly affect facial aesthetics and stability.

Safety has also been confirmed in the literature. Al-Tayar and collaborators, using three-dimensional imaging, observed that sequential distalization with aligners did not induce significant positional or morphological changes in temporomandibular joint structures. Together, these findings suggest that when distalization protocols are carefully individualized and supported by the appropriate auxiliaries, clear aligners can achieve predictable sagittal corrections without compromising vertical stability or joint health.

### 4.5. Determinants of Predictability and the Role of Auxiliaries

Despite these strengths, the overall predictability of complex movements with aligners remains strongly influenced by interrelated factors. Among these, attachment design and placement are particularly crucial. Castroflorio and colleagues highlighted how attachment morphology, such as beveled edges, surface area, and positioning, affects the accuracy of rotations and torque. Similarly, the physical properties of aligner materials, including elasticity, thickness, and resilience, directly influence force delivery and decay over time.

Keilig and collaborators demonstrated that relatively simple movements, such as tipping and linear translations, are generally predictable with aligners. In contrast, more complex displacements—rotations, torque, and vertical control—often deviate substantially from digital plans, producing side effects like anchorage loss or unintended movement of adjacent teeth. This evidence underscores the necessity of overcorrection strategies, staged refinements, and precise monitoring to optimize outcomes.

The adaptability of aligners can, however, be expanded through the incorporation of auxiliaries and adjunctive devices. A remarkable case report by Lu and colleagues described the treatment of a severe hyperdivergent skeletal Class II malocclusion using premolar extractions, temporary anchorage devices (TADs), and intrusion bulbs integrated with aligners. The patient achieved significant incisor and molar intrusion, favorable mandibular rotation, and stable, aesthetically pleasing results that persisted at a four-year follow-up. Such complex cases illustrate how aligners, when combined with appropriate auxiliaries, can reach therapeutic goals previously considered attainable only through fixed appliances or orthognathic surgery.

### 4.6. Broader Considerations and Future Perspectives

Taken together, the literature suggests that clear aligners constitute a versatile and effective treatment modality, offering significant advantages in terms of patient comfort, aesthetics, and specific biomechanical control, particularly in maintaining lower incisor inclination and limiting vertical side effects. Nevertheless, their efficacy in achieving movements like torque, rotations, and true intrusion remains variable and dependent on multiple determinants: the aligner system chosen, the biomechanical strategies employed, the design and placement of attachments, and patient-specific factors, such as bone morphology and compliance.

From a broader perspective, aligner therapy should be understood not as a fixed protocol but as a highly individualized approach. Successful outcomes require a combination of digital planning expertise, deep biomechanical knowledge, and the clinical acumen to recognize when auxiliaries, refinements, or even hybrid approaches with fixed appliances are necessary.

Furthermore, ongoing technological progress promises to enhance aligner performance. Advances in polymer science are leading to the development of materials with improved elastic memory, force delivery, and resistance to deformation. Simultaneously, refinements in digital treatment planning software—especially those incorporating artificial intelligence and machine learning—may increase the accuracy of predicted outcomes and allow for more personalized treatment strategies.

In conclusion, clear aligners represent a clinically robust, adaptable, and patient-centered treatment modality capable of addressing a wide spectrum of malocclusions. Their ability to maintain favorable incisor inclination, provide vertical control in selected scenarios, and deliver enhanced patient comfort and aesthetics makes them a valuable option, particularly for patients who prioritize discretion and comfort. However, their inherent variability in achieving complex movements, such as torque, rotations, and deep bite correction, necessitates meticulous, individualized planning and often the integration of auxiliaries.

The growing body of evidence highlights that aligner therapy is most effective when clinicians fully understand the biomechanical principles involved, carefully evaluate the specific aligner system used, and adopt an evidence-based, critical approach to treatment. With ongoing advancements in materials, design, and digital technologies, it is likely that the predictability and scope of aligner therapy will continue to expand. When applied thoughtfully, aligners offer a sophisticated and flexible orthodontic solution capable of bridging the gap between patient expectations and complex biomechanical demands, thereby shaping the future of contemporary orthodontics.

## 5. Conclusions

The current body of evidence strongly supports clear aligners as an effective and versatile modality for the management of mild to moderate orthodontic corrections and, in select cases, even more complex malocclusions. Across randomized clinical trials, retrospective analyses, and three-dimensional studies, clear aligners have demonstrated reliable control over sagittal and vertical tooth movements, with outcomes that are comparable to, and in some aspects superior to, conventional fixed appliances.

**Figure 2:**
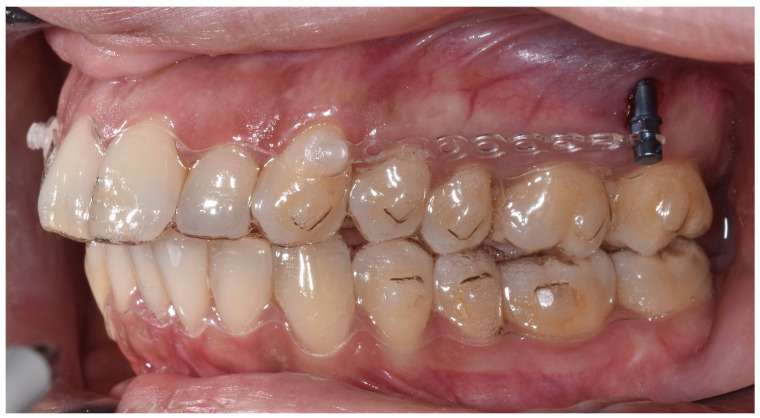
Combined use of aligners and IZC Tads.

**Figure 3:**
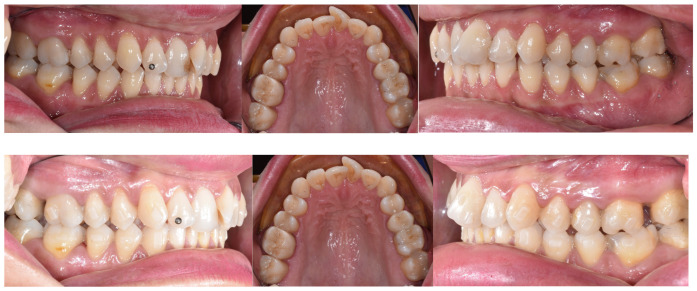
Sequential distalizzation for Class 2 correction and crowding resolution.

The effectiveness of aligners in sagittal correction is particularly evident in Class II distalization protocols, where individualized biomechanics and the strategic use of auxiliary devices significantly enhance predictability (Figure 2). Specifically, the Angel button provided optimal anchorage for central incisors, the precision incision hook supported lateral incisor stability while maximizing molar movement, and the lingual button effectively controlled canine displacement. These findings underscore the importance of tailoring distalization strategies to individual patient characteristics to maximize efficacy and minimize undesirable effects (Figure 3).

Despite these strengths, the literature consistently highlights limitations in the predictability of certain complex movements. Torque, particularly in maxillary central incisors, remains challenging. These findings underscore the necessity of careful system selection when planning complex movements.

Accurate outcomes with aligners also depend on meticulous digital treatment planning, staging strategies, and the incorporation of overcorrection protocols. These insights reinforce the importance of planned overcorrections, ongoing clinical monitoring, and refinement phases to achieve predictable results while minimizing unwanted effects.

The integration of auxiliary devices further enhances the biomechanical potential of aligners. Temporary anchorage devices (TADs), preformed attachments, and elastic traction systems allow for selective intrusions, differential mandibular advancements, and controlled sagittal and vertical movements that would otherwise be difficult to achieve with aligners alone. 

Beyond mechanical control, clear aligners confer notable advantages in patient experience. They reduce soft tissue irritation, facilitate oral hygiene, and improve aesthetic acceptance, factors closely associated with compliance and long-term treatment success. Additionally, their ability to control sagittal and vertical dimensions, particularly in Class II distalization protocols, contributes to favorable skeletal and occlusal outcomes, further supporting their use as a patient-centered alternative to fixed appliances.

The literature also emphasizes the importance of individualized treatment planning and setting realistic expectations. Patient-specific factors, such as age, dental development, craniofacial morphology, and individual biomechanical response, significantly influence treatment efficacy. System-specific variables—including aligner material properties, thickness, and attachment design—interact with these patient characteristics to determine movement precision and predictability. This evidence clearly indicates that aligner therapy should not be applied through a standardized protocol; rather, it requires a tailored approach for each patient. Anticipating potential underexpression of torque, intrusion, and rotation and integrating overcorrection strategies, sequential aligner staging, and refinement phases are critical to achieving clinically satisfactory outcomes.

Furthermore, aligner-mediated distalization and vertical control have minimal impact on temporomandibular joint health. Al-Tayar et al. demonstrated that sequential distalization with aligners produced negligible changes in condylar position or joint morphology, contrasting with certain fixed or removable appliances that can alter mandibular dynamics or induce vertical changes. This highlights the safety profile of aligners and their suitability for non-extraction Class II corrections, reinforcing their reliability in clinical practice.

In conclusion, the synthesis of available studies confirms that clear aligners are an effective, safe, and patient-oriented modality for contemporary orthodontic treatment. They reliably manage sagittal and vertical tooth movements, minimize undesired mandibular incisor proclination, and provide enhanced comfort and aesthetic appeal compared to fixed appliances. While certain complex movements remain challenging, these limitations can be mitigated through careful treatment planning, strategic overcorrection, sequential staging, auxiliary device integration, and refinement phases. With individualized biomechanics and realistic expectations, aligners provide a reliable, versatile, and patient-centered alternative to conventional fixed appliances capable of delivering stable, predictable, and clinically satisfactory outcomes.

## Figures and Tables

**Table 1:** Risk of bias assessment of the included studies.

Studio (Autore, Anno)	D1	D2	D3	D4	D5	D6	D7	D8
J. Hennessy et al. (2016) [5]	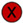 High risk	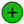 Low risk	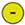 Some concerns	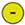 Some concerns	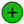 Low risk	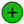 Low risk	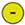 Some concerns	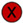 High risk
S. Dianiskova et al. (2022) [34]	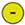 Some concerns	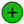 Low risk	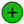 Low risk	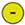 Some concerns	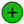 Low risk	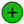 Low risk	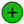 Low risk	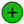 Low risk
R. Lione et al. (2022) [100]	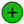 Low risk	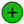 Low risk	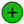 Low risk	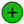 Low risk	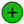 Low risk	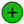 Low risk	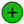 Low risk	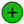 Low risk
A. Balboni et al. (2023) [36]	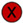 High risk	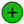 Low risk	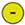 Some concerns	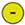 Some concerns	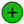 Low risk	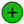 Low risk	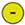 Some concerns	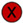 High risk
Y. Li et al. (2025) [99]	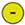 Some concerns	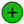 Low risk	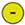 Some concerns	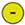 Some concerns	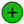 Low risk	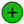 Low risk	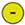 Some concerns	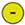 Some concerns
M. Tepedino et al. (2018) [124]	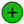 Low risk	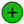 Low risk	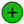 Low risk	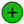 Low risk	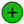 Low risk	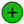 Low risk	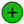 Low risk	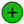 Low risk
W. Lu et al. (2023) [73]	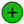 Low risk	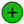 Low risk	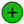 Low risk	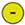 Some concerns	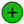 Low risk	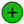 Low risk	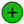 Low risk	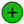 Low risk
T. Castroflorio et al. (2023) [143]	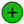 Low risk	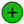 Low risk	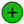 Low risk	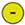 Some concerns	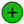 Low risk	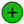 Low risk	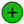 Low risk	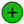 Low risk
B. Al-Tayar et al. (2023) [114]	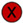 High risk	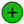 Low risk	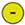 Some concerns	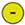 Some concerns	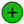 Low risk	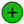 Low risk	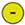 Some concerns	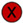 High risk
L. Keilig et al. (2024) [7]	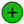 Low risk	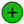 Low risk	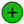 Low risk	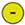 Some concerns	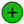 Low risk	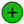 Low risk	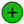 Low risk	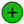 Low risk
N. Shahabuddin et al. (2023) [144]	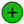 Low risk	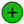 Low risk	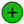 Low risk	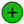 Low risk	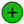 Low risk	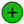 Low risk	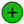 Low risk	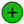 Low risk
N. Rajan et al. (2024) [103]	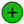 Low risk	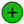 Low risk	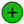 Low risk	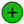 Low risk	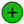 Low risk	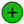 Low risk	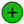 Low risk	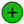 Low risk

Domains: D1: Bias due to confounding. D2: Bias arising from measurement of the exposure. D3: Bias in selection of participants in the study (or the analysis). D4: Bias due to post-exposure interventions. D5: Bias due to missing data. D6: Bias arising from measurement of the outcome. D7: Bias in selection of the reported result. D8: A review-specific extension 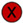 high; 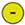 some concerns; 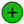 low.

## Data Availability

No new data were created or analyzed in this study. Data sharing is not applicable to this article.

## Abbreviations

Abbreviation	Definition
Class II	Class II (malocclusions according to Angle’s classification)
CAD/CAM	Computer-Aided Design/Computer-Aided Manufacturing
CBCT	Cone Beam Computed Tomography
PRISMA	Preferred Reporting Items for Systematic Reviews and Meta-Analyses
CAT	Clear aligner therapy
CAG	Clear Aligner Group
TADs	Temporary anchorage devices
CAD-CAM	Computer-Aided Design/Computer-Aided Manufacturing
OHRQoL	Oral-Health-Related Quality of Life
OJ	Overjet
OB	Overbite
TMD	Temporomandibular Disorder
CR	Centric Relation
MI	Maximum Intercuspation
VTO	Visual Treatment Objective
G8	Invisalign G8 Protocol
PG	Pendulum Group
IBs	Intrusion bulbs
LII	Little’s Irregularity Index
